# Identifying competing interest disclosures in systematic reviews of surgical interventions and devices: a cross-sectional survey

**DOI:** 10.1186/s12874-020-01144-2

**Published:** 2020-10-19

**Authors:** Jiajie Yu, Guanyue Su, Allison Hirst, Zhengyue Yang, You Zhang, Youping Li

**Affiliations:** 1 Chinese Evidence-based Medicine Center, West China Hospital, Sichuan University, No. 37 Guo Xue Xiang, Chengdu, 610041 Sichuan China; 2 Nuffield Department of Surgical Sciences, University of Oxford, Oxford, OX3 9DU UK; 3 School of Preclinical and Forensic Medicine, Sichuan University, Chengdu, 610041 China; 4 School of Medicine, PanZhiHua University, Panzhihua, 617000 China

**Keywords:** Competing interests, Systematic reviews, Surgical interventions, Devices

## Abstract

**Background:**

A competing interest is an important source of bias in research and disclosure is frequently employed as a strategy to manage it. Considering the importance of systematic reviews (SRs) and the varying prevalence of competing interests in different research fields, we conducted a survey to identify the range of competing interests in SRs assessing surgical interventions or devices and explored the association between the competing interest disclosures and authors’ conclusions.

**Methods:**

We retrieved SRs of surgical interventions and devices published in 2017 via PubMed. Information regarding general characteristics, funding sources, and competing interest disclosures were extracted. We conducted a descriptive analysis of the studies’ characteristics and compared them between Cochrane SRs (CSRs) and non-Cochrane SRs using the Chi-square test. Results were expressed as odds ratio and their 95% confidence interval.

**Results:**

One hundred fifty-five SRs published in 2017 were included in the study. More than half of the SRs (58.7%) reported their funding sources and 94.2% reported authors’ competing interest disclosures. Among 146 SRs that stated competing interest disclosures, only 35 (22.6%) SRs declared at least one author had a competing interest. More than 40 terms were used to describe competing interests. Cochrane SRs (CSRs) were more likely to provide a detailed description of competing interests compared to those in non-CSRs (48.0% versus 25.4%, *P* = 0.023). No association between positive conclusions and competing interest disclosures was found (*P* = 0.484, OR = 0.43, 95%CI: 0.08, 2.16). In the subgroup analyses, SRs stating no competing interest disclosure were more likely to report positive conclusions than those stating at least one type of competing interest, but the difference is not significantly different (*P* = 0.406, OR = 1.38, 95%CI: 0.64, 2.98).

**Conclusion:**

In surgical SRs, there is a high percentage of competing interest disclosures but without detailed information. The identification and statement of competing interests with a detailed description, particularly the non-financial ones, needs improvement. Some efficient and effective methods/tools for identifying, quantifying, and minimizing potential competing interests in systematic reviews remains valuable.

## Background

A competing interest is defined as “a set of circumstances which create a risk that professional judgment or actions regarding a primary interest will be unduly influenced by a secondary interest” [1, 2] and considered to be “ubiquitous and inevitable in academic life” [3, 4]. A full disclosure is acknowledged as an important method for reporting and managing competing interests and serves to highlight the potential for bias [5–8]. Since 1988, the International Committee of Medical Journal Editors (ICMJE) has required authors to disclose financial or other relationships that might lead to a competing interest [9], and hundreds of other biomedical journals put similar policy into practice [10].

The number of systematic reviews (SR) increased rapidly in the recent year for providing end-users with a comprehensive, critical, and up to date method to synthesize available evidence. Meanwhile, studies regarding competing interests of systematic reviews increased for the concerns about suboptimal decisions made by end-users for potential competing interests [11–16]. Surgery, with its wide use of medical devices and strong personal preference for interventions, could be a field with high potential for competing interests [17]. In 2017, the JAMA published a collection to discuss issues relating to competing interests and stated that transparent disclosure is increasingly emphasized as an essential component in the reporting of both clinical trials and systematic reviews [18, 19]. Although numerous published studies addressed competing interests in medical research, there is a significant disproportion in the number of studies assessing competing interests, especially non-financial interests in surgical interventions and devices research [20–22].

Considering the importance of SRs and the varying prevalence of competing interests in different fields [23]. We, therefore, performed a survey to identify the range of competing interests in systematic reviews assessing surgical interventions and devices and proposed a potential checklist for competing interest disclosures in surgical research. We also explored the impact of competing interest disclosures on the study conclusions.

## Methods

### Eligibility criteria

A study was included if it was described as a systematic review or a meta-analysis and only included RCTs assessing at least one surgical intervention or device. Network meta-analyses, methodological systematic reviews or systematic reviews reported as conference abstracts and research letters were excluded. A systematic review was defined as described in the Cochrane handbook (a. a clearly stated set of objectives with an explicit, reproducible methodology; b. a systematic search that attempts to identify all studies that would meet the eligibility criteria; c. an assessment of the validity of the findings of the included studies, such as through the assessment of risk of bias; and d. systematic presentation and synthesis of the characteristics and findings of the included studies) [24] and the definitions of a surgical intervention and device have been described elsewhere [25]. A competing interest disclosure was defined as whether a competing interest disclosure was stated or not.

### Data resource and study procedures

Systematic reviews were identified through PubMed, we limited our search to literature published in the English language, and between 1 January 2017 and 31 December 2017 due to a massive number of published surgical studies. The search strategy was based on MeSH terms and their variants and developed in collaboration with an experienced librarian (Additional file 1). Two reviewers (JY and GS), trained in trial and systematic review methods, used predefined, pilot-tested forms independently to screen abstracts and full texts for eligibility.

### Data collection

Two teams of reviewers (JY and GS, YZ and ZY) independently collected data from eligible studies. Twenty systematic reviews met eligibility criteria were enrolled for a pilot study before the formal screening and data extraction. We iteratively adjusted the screening rules to improve the consistency between reviewers. Any conflicts not resolved by discussion between the reviewers were referred to the study team. Coher’s κ statistic was used to assess the concordance of reviewers, and a value of 0.75 or greater was chosen for satisfactory agreement.

The following general information was collected for each eligible study: (1) number of authors; (2) country of the corresponding author; (3) number of trials included; (4) total number of participants involved; (5) review type (Cochrane SR or non-Cochrane SR); (6) adherence to PRISMA (Preferred Reporting Items for Systematic Reviews and Meta-Analyses); (7) type of journal (general or surgical journal); (8) type of control (surgical intervention, non-surgical intervention or both); (9) involvement of methodologist (i.e. statistician, epidemiologist). We judged that a systematic review involved a methodologist if any of the authors were affiliated with a department of epidemiology, statistics, or evidence-based medicine, or listed in the acknowledgement section.

We extracted the funding sources and classified them as industry funding (e.g. funding from the device industry; company; insurance company), non-industry funding (e.g. funding from the academic institution; university; hospital; government bodies; foundation; charity et al.), no funding and not reported. We extracted the statement of competing interests verbatim and then categorized them based on their characteristics and nature.

The authors’ conclusion was labelled “positive” when the overall results favoured intervention over control or both groups were equal but the interventional group had minor advantages. A “negative” conclusion was defined if the results favoured control or both groups were equal [26].

### Data analysis

We conducted a descriptive analysis of study characteristics of included systematic reviews. For all descriptive analyses, frequencies (and percentages) were used for dichotomous variables, and mean (and standard deviation) or median (and range) for continuous variables. We compared the funding sources and competing interest disclosures between Cochrane SR and non-CSR using Fisher’s exact test for dichotomous variables.

We categorized the studies into two groups based on whether the investigators disclosed competing interests or not. If they did, we then divided the competing interest disclosure group into two subgroups (at least one type of competing interest was disclosed and no competing interest disclosure group). The differences in the authors’ conclusions were compared between groups using the Chi-square test. Results were expressed as odds ratios and their 95% confidence interval. All analyses were performed by IBM SPSS Statistics 26.

## Results

**Fig. 1:**
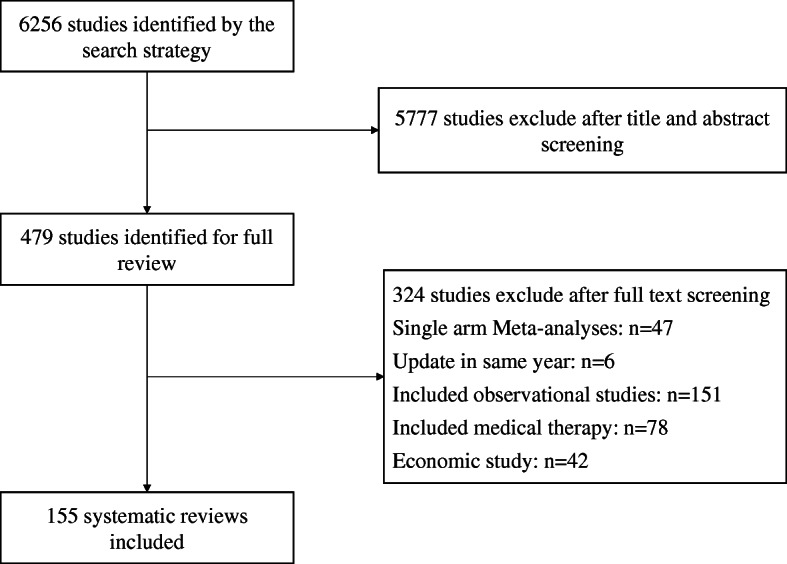
Study selection

A total of 6256 systematic reviews were identified from PubMed. After the title and abstract screening, 479 were found to be potentially eligible, of which 155 were finally included based on the full-text article review (Fig. 1). The interobserver agreement on data collection was good (κ = 0.84). A full list of the included SRs is presented in Additional file 2.

### General information from the included SRs

The general characteristics are listed in Table 1. The median number of included trials was 6 (range 2 to 21), 25 (16.1%) were Cochrane SRs and 89 (57.4%) mentioned PRISMA in their studies. 109 (70.3%) were published in general medical journals; 23 (16.1%) involved researchers with affiliations with either epidemiology or statistics departments, and 132 (85.2%) tested alternative surgical interventions.

**Table 1:** General characteristics of included systematic review

Characteristics	*n* = 155
No of authors ^a^	6 (2–21)
No of study included ^a^	7 (1–44)
No of participants included ^a^	1167 (80–19,886)
No of database included ^a^	4 (1–15)
Methodologist involved	23 (14.8)
Cochrane SR	25 (16.1)
PRISMA mentioned	89 (57.4)
Type of journal	
General	109 (70.3)
Surgical	46 (29.7)
Country of corresponded author	
China	48 (31.0)
USA	22 (14.2)
UK	18 (11.6)
Italy	12 (7.7)
Australia	12 (7.7)
Other	43 (27.8)
Type of comparison	
Surgical versus surgical	132 (85.2)
Surgical versus non-surgical	17 (10.9)
Surgical versus both	6 (3.9)
Comparisons between different surgical procedures	
Procedures	79 (59.8)
Devices	53 (40.2)
Specialty	
General	54 (34.9)
Cardiothoracic	45 (29.0)
Orthopedic	24 (15.5)
Other	32 (20.6)

Values in parentheses are percentages unless indicated otherwise

^a^ values are median (range)

### Funding information from the included SRs

Of the 155 selected SRs, 42 (27.1%) reported review funding from governmental agencies and academic institutions, three (1.9%) reported review funding from pharmaceutical or devices company, 45 (29.0%) stated the reviews were not funded; and 64 (41.3%) did not provide the funding information (Table 2).

**Table 2:** Classification of funding source and competing interests in the included systematic reviews

	Overall (*n* = 155)	CSR (*n* = 25)	Non-CSR (*n* = 130)
Source of funding			
Industry funding	3 (1.9)	0 (0)	3 (2.3)
Non-industry funding	42 (27.1)	20 (80.0)	22 (16.9)
Industry + non-industry funding	1 (0.6)	0 (0)	1 (0.8)
Reported as not funded	45 (29.0)	1 (4.0)	44 (33.8)
Not reported	64 (41.3)	4 (16.0)	60 (46.2)
Types of competing interests			
No competing interest to disclose	111 (71.6)	12 (48.0)	99 (76.2)
At least one type			
Grant/fellowship	17 (11.0)	6 (24.0)	11 (8.5)
Honoraria	15 (9.7)	2 (8.0)	13 (1.0)
Consulting	14 (9.0)	4 (16.0)	10 (7.7)
Non-monetary support	8 (5.2)	5 (20.0)	3 (2.3)
Service in other affiliations	8 (5.2)	2 (8.0)	6 (4.6)
Equity/stocks/bonds	3 (1.9)	1 (4.0)	2 (1.5)
Patent	2 (1.3)	0 (0)	2 (1.5)
Employment/salary	1 (0.6)	1 (4.0)	0 (0)
Leadership in company	1 (0.6)	0 (0)	1 (0.8)
Intellectual beliefs	6 (3.8)	4 (16.0)	2 (1.5)
Experience	3 (1.9)	2 (8.0)	1 (0.8)
Personal relationship	2 (1.3)	2 (8.0)	0 (0)

Values in parentheses are percentages

*CSR* Cochrane systematic review

### Competing interest disclosures concerning the included SRs

One hundred and forty-six studies (94.2%) disclosed the information of authors’ competing interests. However, of the 146 systematic reviews that provided competing interest disclosures, only 35 (22.6%) SRs declared at least one author had one competing interest. More than 40 terms were used to describe competing interests, and we categorized them into 12 terms that were the most common descriptions of competing interests, including grants/fellowship, honoraria, consulting, employment/salary, patent/copyright, equity/stocks/bonds, non-monetary support, service in other affiliations, founder or other leadership in a company, intellectual beliefs, experience, and personal relationship.

Of the 35 SRs that stated competing interests, Cochrane SRs were more likely to provide a detail description of the competing interests in comparison to those that are non-CSRs (48.0% versus 25.4%, *P* = 0.023). The most frequently reported types was “grant/fellowship” (*n* = 17, 11.0%), followed by “honoraria” (*n* = 15, 9.7%), “consulting” (*n* = 14, 9.0%), “non-monetary support” and “services in other affiliations” (*n* = 8, 5.2%) (Table 2).

We found no significant difference between SRs that had a competing interest disclosure and those that did not (*P* = 0.484, OR = 0.43, 95%CI: 0.08, 2.16). In the subgroup analyses, SRs stating no competing interest disclosure were more likely to report a positive conclusion than those stating at least one type of competing interest. However, the difference is still not significant (*P* = 0.406, OR = 1.38, 95%CI: 0.64, 2.98).

## Discussion

Our survey shows that 94.2% of surgical systematic reviews published in 2017 reported authors’ competing interest disclosures. This result is in line with the strict journal requirement for more than 95% of medical journals have a policy, that refers to competing interest disclosure as an essential part of biomedical studies [16]. However, of the147 studies that declared their competing interests, only 35 (22.6%) provided a detailed disclosure.

There are several possible reasons for the low rate of detailed competing interest disclosures. One of the reasons is, most researchers are unaware of the fact that behaviours which include receiving support for food and beverages or academic competition are also competing interests and could affect their research [9, 27]. Another potential possibility is the policies regarding some competing interests are regularly obscure, particularly non-financial competing interests [16, 28–30]. We reviewed the “instruction to authors” on journals’ websites and found that the instructions of competing interests are limited, with most of them only involving a few types, such as a grant, consulting fee and employment. We have summarized a checklist of competing interests based on our results to assist researchers in identifying potential competing interests. Journals should also consider to include this checklist in their policies or instructions (Table 3).

**Table 3:** A potential checklist of competing interest disclosure

Proposed terms	Descriptions
***Financial competing interests***	
Personal fees/payment	Fees paid to person for consulting, lecturer, speakers bureaus, expert testimony, presentations, manuscript preparation, educational support, writing and reviewing assistant
Employment/salary	The professional is/was employed by a company and a periodic payment is/was provided by the company
Non-monetary support	Travel paid, accommodations, meeting expenses, administrative support; food; beverage
Drug/equipment supplies	The provision of surgical or research devices by a company
Grant	Grant from a company, or governmental agency, hospital, university or other institutions
Patent(s)/copyright	The professional holds or shares a patent
Equity/stocks/bonds	Ownership of interest in a company
***Non-financial competing interests***	
Service in other affiliates	Such as scientific advisory board, steering committee membership
Leadership in a company	The professional holds the position of a high ranking corporate officer in a company and has responsibility for its operation
Intellectual beliefs	Authorship of primary studies included or not include in the SR; Participation in a previous guideline panel or editorial
Faith and fixed beliefs	Such as political beliefs, religious beliefs, culture practices and dietary habits
Personal experience	Specialty training; experience with specific population; personal preference
Personal relationship	Social relationship
Academic competition	Driven by some competitive academic or desire for glory

Several methodological studies have been conducted to assess the reporting of competing interest disclosure, and there is substantial variability in the reporting rates among different study designs and specialties (17.0 to 71.2%) [16–19, 21, 22, 31–34]. Generally, authors of randomized controlled trials and cohort studies more likely to provide detailed information rather than those in systematic reviews. Our finding on the rate of competing interest disclosure is consistent with other studies assessing SRs [16–18] and no significant difference was found between trials in the drug and surgical field.

A competing interest in biomedical research has often been associated with a positive conclusion [19, 20, 35–37]; however, we did not have a similar finding in our study. Considering Cochrane’ policy regarding funding and competing interest is stricter than other journals [27], we also conducted sensitivity analyses that only included non-CSRs and the result was the same. Notably, our findings should be cautiously interpreted for the low percentage of SRs sponsored by industries in comparison to other published studies [19, 35].

We used a systematic method for data screening and extraction and provided a potential checklist of competing interests to readers and editors based on the results. However, our study also has some limitations. First, the restriction of our search to one database and year could compromise the generalizability of findings. Second, these disclosures have all been reported by authors, and we have limited methods to confirm or verify them. Thirdly, we only assessed SRs that included randomized controlled trials which could have underestimated the rate of competing interest disclosures.

## Conclusions

In summary, 94% of SRs assessing surgical interventions and devices stated their competing interest disclosures but without detailed information. The identification and statement of competing interests, particularly non-financial interests, remains challenging for researchers, reviewers, and editors. The International Committee of Medical Journal Editors (ICMJE) and other communities need to continue to develop more efficient and effective methods/tools for identifying, quantifying, and minimizing potential competing interests in systematic reviews.

## Supplementary information

**Additional file 1.** Appendix Search strategy.

**Additional file 2.** A full lists of included SRs.

## Data Availability

The dataset supporting the conclusions of this article are included within the article and its additional files.

## Abbreviations

SR: Systematic review
ICMJE: International Committee of Medical Journal Editors
CSRs: Cochrane systematic review
