# Supplementary material for: Identifying competing interest disclosures in systematic reviews of surgical interventions and devices: a cross-sectional survey
Source: BMC Med Res Methodol. 2020 Oct 19;20:260. doi: 10.1186/s12874-020-01144-2 (PMC7574563; doi:10.1186/s12874-020-01144-2)
Supplement: Supplementary file 1 — Additional file 1. Appendix Search strategy. [file 12874_2020_1144_MOESM1_ESM.docx]

**Appendix Search strategy**

**PubMed:**

#1 "Specialties, Surgical"[Mesh]

#2 "Surgical Procedures, Operative"[Mesh]

#3 "Surgery" [Subheading]

#4 Surgery [tw] OR surgeries [tw] OR surgerys [tw]

#5 Operation [tw] OR operations [tw]

#6 Surgical procedure*[tw] OR Surgical operation*[tw] OR Surgical treatment*[tw] OR Surgical therap*[tw] OR Surgical intervention*[tw] OR Surgical research*[tw]

#7 Operative procedure*[tw] OR Operative operation*[tw] OR Operative treatment*[tw] OR Operative therap*[tw] OR Operative intervention*[tw] OR Operative research*[tw] Invasive procedure*[tw] OR Invasive intervention*[tw]

#8 Peroperative procedure*[tw] OR Peroperative intervention*[tw] OR Perioperative procedure*[tw] OR Perioperative intervention*[tw] OR Preoperative procedure*[tw] OR Preoperative intervention*[tw] OR Intraoperative procedure*[tw] OR Intraoperative intervention*[tw]

#8 #1 OR #2 OR #3 OR #4 OR #5 OR #6 OR #7

#9 "Review Literature as Topic"[Mesh] OR "Meta-Analysis as Topic"[Mesh]

#10 "Meta-Analysis" [Publication Type]

#11 Meta-analy*[tw] OR meta analy*[tw] OR Metaanaly*[tw] OR systematic review*[tw]

#12 Cochrane[tiab] OR Embase[tiab] OR medline[tiab] OR pubmed[tiab] OR medlars[tiab]

#13 Psychlit[tiab] OR psyclit[tiab] OR Cinahl[tiab] OR cinhal[tiab] OR Science citation index[tiab] OR Bids[tiab] OR Cancerlit[tiab]

#14 Reference list*[tiab] OR bibliograph*[tiab] OR Hand-search*[tiab] OR Relevant journals[tiab] OR Manual search*[tiab]

#15 Selection criteria[tiab] OR data extraction[tiab]) AND Review[Publication Type]

#16 Comment[Publication Type] OR letter[Publication Type] OR editorial[Publication Type]

#17 #9 OR #10 OR #11 OR #12 OR #13 OR #14 OR #15 OR #16

#18 #8 AND #17

#19 animals [Mesh]

#20 humans [Mesh]

#21 #19 NOT #20

#22 #18 NOT #21
