# Supplementary material for: Identifying competing interest disclosures in systematic reviews of surgical interventions and devices: a cross-sectional survey
Source: BMC Med Res Methodol. 2020 Oct 19;20:260. doi: 10.1186/s12874-020-01144-2 (PMC7574563; doi:10.1186/s12874-020-01144-2)
Supplement: Supplementary file 2 — Additional file 2. A full lists of included SRs. [file 12874_2020_1144_MOESM2_ESM.docx]

Additional file A full lists of included SRs

1. Abraha I, Binda GA, Montedori A, Arezzo A, Cirocchi R. Laparoscopic versus open resection for sigmoid diverticulitis. Cochrane Database Syst Rev. 2017;11(doi):CD009277.
2. Agarwal N, Jain A, Garg J, Mojadidi MK, Mahmoud AN, Patel NK, et al. Staged versus index procedure complete revascularization in ST-elevation myocardial infarction: A meta-analysis. J Interv Cardiol. 2017;30(5):397-404.
3. Al Otaibi A, Gupta S, Belley-Cote EP, Alsagheir A, Spence J, Parry D, et al. Mini-thoracotomy vs. conventional sternotomy mitral valve surgery: a systematic review and meta-analysis. J Cardiovasc Surg (Torino). 2017;58(3):489-96.
4. Alcelik I, Blomfield M, Ozturk C, Soni A, Charity R, Acornley A. A comparison of short term radiological alignment outcomes of the patient specific and standard instrumentation for primary total knee arthroplasty: A systematic review and meta-analysis. Acta Orthop Traumatol Turc. 2017;51(3):215-22.
5. Allievi N, Ceresoli M, Fugazzola P, Montori G, Coccolini F, Ansaloni L. Endoscopic Stenting as Bridge to Surgery versus Emergency Resection for Left-Sided Malignant Colorectal Obstruction: An Updated Meta-Analysis. Int J Surg Oncol. 2017;2017(19-20):2863272.
6. Anderson PA, Nassr A, Currier BL, Sebastian AS, Arnold PM, Fehlings MG, et al. Evaluation of Adverse Events in Total Disc Replacement: A Meta-Analysis of FDA Summary of Safety and Effectiveness Data. Global Spine J. 2017;7(1 Suppl):76S-83S.
7. Antoniou GA, Georgiadis GS, Antoniou SA, Makar RR, Smout JD, Torella F. Bypass surgery for chronic lower limb ischaemia. Cochrane Database Syst Rev. 2017;4(3):CD002000.
8. Antoniou SA, Mavridis D, Hajibandeh S, Hajibandeh S, Antoniou GA, Gorter R, et al. Optimal stump management in laparoscopic appendectomy: A network meta-analysis by the Minimally Invasive Surgery Synthesis of Interventions and Outcomes Network. Surgeon. 2018;16(2):119-24. doi: 10.1016/j.surge.2017.07.005. Epub Aug 31.
9. Arezzo A, Passera R, Lo Secco G, Verra M, Bonino MA, Targarona E, et al. Stent as bridge to surgery for left-sided malignant colonic obstruction reduces adverse events and stoma rate compared with emergency surgery: results of a systematic review and meta-analysis of randomized controlled trials. Gastrointest Endosc. 2017;86(3):416-26.
10. Bavishi C, Baber U, Panwar S, Pirrotta S, Dangas GD, Moreno P, et al. Efficacy and safety of everolimus and zotarolimus-eluting stents versus first-generation drug-eluting stents in patients with diabetes: A meta-analysis of randomized trials. Int J Cardiol. 2017;230(2):310-8.
11. Bravo CA, Hirji SA, Bhatt DL, Kataria R, Faxon DP, Ohman EM, et al. Complete versus culprit-only revascularisation in ST elevation myocardial infarction with multi-vessel disease. Cochrane Database Syst Rev. 2017;5(Suppl 2):CD011986.
12. Bundhun PK, Yanamala CM, Huang WQ. Comparing Stent Thrombosis associated with Zotarolimus Eluting Stents versus Everolimus Eluting Stents at 1 year follow up: a systematic review and meta-analysis of 6 randomized controlled trials. BMC Cardiovasc Disord. 2017;17(1):84.
13. Burrage M, Moore P, Cole C, Cox S, Lo WC, Rafter A, et al. Transcatheter Aortic Valve Replacement is Associated with Comparable Clinical Outcomes to Open Aortic Valve Surgery but with a Reduced Length of In-Patient Hospital Stay: A Systematic Review and Meta-Analysis of Randomised Trials. Heart Lung Circ. 2017;26(3):285-95.
14. Carson-Chahhoud KV, Wakai A, van Agteren JE, Smith BJ, McCabe G, Brinn MP, et al. Simple aspiration versus intercostal tube drainage for primary spontaneous pneumothorax in adults. Cochrane Database Syst Rev. 2017;9(1):CD004479.
15. Chapman SJ, Wood B, Drake TM, Young N, Jayne DG. Systematic Review and Meta-analysis of Prophylactic Mesh During Primary Stoma Formation to Prevent Parastomal Hernia. Dis Colon Rectum. 2017;60(1):107-15.
16. Charoenkwan K, Iheozor-Ejiofor Z, Rerkasem K, Matovinovic E. Scalpel versus electrosurgery for major abdominal incisions. Cochrane Database Syst Rev. 2017;6(1):CD005987.
17. Charoenkwan K, Kietpeerakool C. Retroperitoneal drainage versus no drainage after pelvic lymphadenectomy for the prevention of lymphocyst formation in women with gynaecological malignancies. Cochrane Database Syst Rev. 2017;6(6):CD007387.
18. Chen BL, Guo JB, Zhang HW, Zhang YJ, Zhu Y, Zhang J, et al. Surgical versus non-operative treatment for lumbar disc herniation: a systematic review and meta-analysis. Clin Rehabil. 2018;32(2):146-60.
19. Cheng JW, Li Y, Xing WQ, Lv HW, Wang HR. Laparoscopic Heller myotomy is not superior to pneumatic dilation in the management of primary achalasia: Conclusions of a systematic review and meta-analysis of randomized controlled trials. Medicine (Baltimore). 2017;96(7):e6114. doi: 10.1097/MD.0000000000006114.
20. Cheng Y, Briarava M, Lai M, Wang X, Tu B, Cheng N, et al. Pancreaticojejunostomy versus pancreaticogastrostomy reconstruction for the prevention of postoperative pancreatic fistula following pancreaticoduodenectomy. Cochrane Database Syst Rev. 2017;9(1):CD012257.
21. Cheng Y, Xiong X, Lu J, Wu S, Zhou R, Cheng N. Early versus delayed appendicectomy for appendiceal phlegmon or abscess. Cochrane Database Syst Rev. 2017;6(26):CD011670.
22. Ciccotti MC, Secrist E, Tjoumakaris F, Ciccotti MG, Freedman KB. Anatomic Anterior Cruciate Ligament Reconstruction via Independent Tunnel Drilling: A Systematic Review of Randomized Controlled Trials Comparing Patellar Tendon and Hamstring Autografts. Arthroscopy. 2017;33(5):1062-71 e5.
23. Cirocchi R, Di Saverio S, Weber DG, Tabola R, Abraha I, Randolph J, et al. Laparoscopic lavage versus surgical resection for acute diverticulitis with generalised peritonitis: a systematic review and meta-analysis. Tech Coloproctol. 2017;21(2):93-110.
24. Cohen R, Le Roux CW, Junqueira S, Ribeiro RA, Luque A. Roux-En-Y Gastric Bypass in Type 2 Diabetes Patients with Mild Obesity: a Systematic Review and Meta-analysis. Obes Surg. 2017;27(10):2733-9.
25. Collet C, Asano T, Miyazaki Y, Tenekecioglu E, Katagiri Y, Sotomi Y, et al. Late thrombotic events after bioresorbable scaffold implantation: a systematic review and meta-analysis of randomized clinical trials. Eur Heart J. 2017;38(33):2559-66.
26. Coppolino G, Pisano A, Rivoli L, Bolignano D. Renal denervation for resistant hypertension. Cochrane Database Syst Rev. 2017;2(1):CD011499.
27. Cornille JB, Pathak S, Daniels IR, Smart NJ. Prophylactic mesh use during primary stoma formation to prevent parastomal hernia. Ann R Coll Surg Engl. 2017;99(1):2-11.
28. Cross AJ, Buchwald PL, Frizelle FA, Eglinton TW. Meta-analysis of prophylactic mesh to prevent parastomal hernia. Br J Surg. 2017;104(3):179-86.
29. Cui K, Lyu S, Song X, Yuan F, Xu F, Zhang M, et al. Drug-eluting balloon versus bare-mental stent and drug-eluting stent for de novo coronary artery disease: A systematic review and meta-analysis of 14 randomized controlled trials. PLoS One. 2017;12(4):e0176365.
30. Dahal K, Rijal J, Shahukhal R, Sharma S, Watti H, Azrin M, et al. Comparison of manual compression and vascular hemostasis devices after coronary angiography or percutaneous coronary intervention through femoral artery access: A meta-analysis of randomized controlled trials. Cardiovasc Revasc Med. 2018;19(2):151-62.
31. Dai L, Shuai J. Laparoscopic versus open appendectomy in adults and children: A meta-analysis of randomized controlled trials. United European Gastroenterol J. 2017;5(4):542-53.
32. De Luca G, Smits P, Hofma SH, Di Lorenzo E, Vlachojannis GJ, Van't Hof AWJ, et al. Everolimus eluting stent vs first generation drug-eluting stent in primary angioplasty: A pooled patient-level meta-analysis of randomized trials. Int J Cardiol. 2017;244(doi):121-7.
33. De Rosa S, Polimeni A, Sabatino J, Indolfi C. Long-term outcomes of coronary artery bypass grafting versus stent-PCI for unprotected left main disease: a meta-analysis. BMC Cardiovasc Disord. 2017;17(1):240.
34. Deng L, Xiong J, Xia Q. Single-incision versus conventional three-incision laparoscopic appendectomy: A meta-analysis of randomized controlled trials. J Evid Based Med. 2017;10(3):196-206.
35. Deng S, Sun Z, Zhang C, Chen G, Li J. Surgical Treatment Versus Conservative Management for Acute Achilles Tendon Rupture: A Systematic Review and Meta-Analysis of Randomized Controlled Trials. J Foot Ankle Surg. 2017;56(6):1236-43.
36. Devitt BM, Bell SW, Webster KE, Feller JA, Whitehead TS. Surgical treatments of cartilage defects of the knee: Systematic review of randomised controlled trials. Knee. 2017;24(3):508-17.
37. Diniz JM, Botelho RV. Is fusion necessary for thoracolumbar burst fracture treated with spinal fixation? A systematic review and meta-analysis. J Neurosurg Spine. 2017;27(5):584-92.
38. Dong L, Xu Z, Chen X, Wang D, Li D, Liu T, et al. The change of adjacent segment after cervical disc arthroplasty compared with anterior cervical discectomy and fusion: a meta-analysis of randomized controlled trials. Spine J. 2017;17(10):1549-58.
39. Dressler J, Jorgensen LN. The use of expanding ports in laparo-endoscopic single-site surgery may cause more pain: a meta-analysis of randomized clinical trials. Surg Endosc. 2017;31(11):4400-11.
40. Du X, Wu JM, Hu ZW, Wang F, Wang ZG, Zhang C, et al. Laparoscopic Nissen (total) versus anterior 180 degrees fundoplication for gastro-esophageal reflux disease: A meta-analysis and systematic review. Medicine (Baltimore). 2017;96(37):e8085.
41. El-Hayek G, Bangalore S, Casso Dominguez A, Devireddy C, Jaber W, Kumar G, et al. Meta-Analysis of Randomized Clinical Trials Comparing Biodegradable Polymer Drug-Eluting Stent to Second-Generation Durable Polymer Drug-Eluting Stents. JACC Cardiovasc Interv. 2017;10(5):462-73.
42. Emile SH, Elfeki H. Desarda's technique versus Lichtenstein technique for the treatment of primary inguinal hernia: a systematic review and meta-analysis of randomized controlled trials. Hernia. 2018;22(3):385-95.
43. Fan T, Li Y, Deng WW, Wu T, Zhang W. Short Implants (5 to 8 mm) Versus Longer Implants (>8 mm) with Sinus Lifting in Atrophic Posterior Maxilla: A Meta-Analysis of RCTs. Clin Implant Dent R. 2017;19(1):207-15.
44. Farag S, Rehman S, Sains P, Baig MK, Sajid MS. Early vs delayed closure of loop defunctioning ileostomy in patients undergoing distal colorectal resections: an integrated systematic review and meta-analysis of published randomized controlled trials. Colorectal Dis. 2017;19(12):1050-7.
45. Fergo C, Burcharth J, Pommergaard HC, Kildebro N, Rosenberg J. Three-dimensional laparoscopy vs 2-dimensional laparoscopy with high-definition technology for abdominal surgery: a systematic review. Am J Surg. 2017;213(1):159-70.
46. Frost JA, Webster KE, Bryant A, Morrison J. Lymphadenectomy for the management of endometrial cancer. Cochrane Database Syst Rev. 2017;10(8):CD007585.
47. Gaffar R, Habib B, Filion KB, Reynier P, Eisenberg MJ. Optimal Timing of Complete Revascularization in Acute Coronary Syndrome: A Systematic Review and Meta-Analysis. J Am Heart Assoc. 2017;6(4):10.1155/2017/7075935. Epub 2017 Mar 15.
48. Gao K, Sun Y, Yang M, Han L, Chen L, Hu W, et al. Efficacy and safety of polymer-free stent versus polymer-permanent drug-eluting stent in patients with acute coronary syndrome: a meta-analysis of randomized control trials. BMC Cardiovasc Disord. 2017;17(1):194.
49. Gao L, Liu Y, Sun Z, Wang Y, Cao F, Chen Y. Percutaneous coronary intervention using drug-eluting stents versus coronary artery bypass graft surgery in left main coronary artery disease an updated meta-analysis of randomized clinical trials. Oncotarget. 2017;8(39):66449-57.
50. Gao YC, Chen J, Qin Q, Chen H, Wang W, Zhao J, et al. Efficacy and safety of laparoscopic bile duct exploration versus endoscopic sphincterotomy for concomitant gallstones and common bile duct stones: A meta-analysis of randomized controlled trials. Medicine (Baltimore). 2017;96(37):e7925.
51. Garg A, Rao SV, Agrawal S, Theodoropoulos K, Mennuni M, Sharma A, et al. Meta-Analysis of Randomized Controlled Trials of Percutaneous Coronary Intervention With Drug-Eluting Stents Versus Coronary Artery Bypass Grafting in Left Main Coronary Artery Disease. Am J Cardiol. 2017;119(12):1942-8.
52. Giacoppo D, Colleran R, Cassese S, Frangieh AH, Wiebe J, Joner M, et al. Percutaneous Coronary Intervention vs Coronary Artery Bypass Grafting in Patients With Left Main Coronary Artery Stenosis: A Systematic Review and Meta-analysis. JAMA Cardiol. 2017;2(10):1079-88.
53. Gimzewska M, Jackson AI, Yeoh SE, Clarke M. Totally percutaneous versus surgical cut-down femoral artery access for elective bifurcated abdominal endovascular aneurysm repair. Cochrane Database Syst Rev. 2017;2(2):CD010185.
54. Glazener CM, Cooper K, Mashayekhi A. Anterior vaginal repair for urinary incontinence in women. Cochrane Database Syst Rev. 2017;7(6):CD001755.
55. Gozdek M, Pawliszak W, Hagner W, Zalewski P, Kowalewski J, Paparella D, et al. Systematic review and meta-analysis of randomized controlled trials assessing safety and efficacy of posterior pericardial drainage in patients undergoing heart surgery. J Thorac Cardiovasc Surg. 2017;153(4):865-75 e12.
56. Grant MC, Yang D, Wu CL, Makary MA, Wick EC. Impact of Enhanced Recovery After Surgery and Fast Track Surgery Pathways on Healthcare-associated Infections: Results From a Systematic Review and Meta-analysis. Ann Surg. 2017;265(1):68-79.
57. Hao XY, Shen YF, Wei YG, Liu F, Li HY, Li B. Safety and effectiveness of day-surgery laparoscopic cholecystectomy is still uncertain: meta-analysis of eight randomized controlled trials based on GRADE approach. Surgical Endoscopy and Other Interventional Techniques. 2017;31(12):4950-63.
58. Haueter R, Schutz T, Raptis DA, Clavien PA, Zuber M. Meta-analysis of single-port versus conventional laparoscopic cholecystectomy comparing body image and cosmesis. Br J Surg. 2017;104(9):1141-59.
59. Holihan JL, Hannon C, Goodenough C, Flores-Gonzalez JR, Itani KM, Olavarria O, et al. Ventral Hernia Repair: A Meta-Analysis of Randomized Controlled Trials. Surg Infect (Larchmt). 2017;18(6):647-58.
60. Huo ZC, Liu G, Li XY, Liu F, Fan WJ, Guan RH, et al. Use of a disposable circumcision suture device versus conventional circumcision: a systematic review and meta-analysis. Asian Journal of Andrology. 2017;19(3):362-7.
61. Huttner FJ, Probst P, Knebel P, Strobel O, Hackert T, Ulrich A, et al. Meta-analysis of prophylactic abdominal drainage in pancreatic surgery. Br J Surg. 2017;104(6):660-8.
62. Ilic D, Evans SM, Allan CA, Jung JH, Murphy D, Frydenberg M. Laparoscopic and robotic-assisted versus open radical prostatectomy for the treatment of localised prostate cancer. Cochrane Database Syst Rev. 2017;9(5):CD009625.
63. Jin B, Chen MT, Fei YT, Du SD, Mao YL. Safety and efficacy for laparoscopic versus open hepatectomy: A meta-analysis. Surg Oncol. 2018;27(2):A26-A34.
64. Kakkos SK, Kakisis I, Tsolakis IA, Geroulakos G. Endarterectomy achieves lower stroke and death rates compared with stenting in patients with asymptomatic carotid stenosis. J Vasc Surg. 2017;66(2):607-17.
65. Kallidonis P, Ntasiotis P, Knoll T, Sarica K, Papatsoris A, Somani BK, et al. Minimally Invasive Surgical Ureterolithotomy Versus Ureteroscopic Lithotripsy for Large Ureteric Stones: A Systematic Review and Meta-analysis of the Literature. Eur Urol Focus. 2017;3(6):554-66.
66. Khan AR, Golwala H, Tripathi A, Riaz H, Kumar A, Flaherty MP, et al. Meta-analysis of Percutaneous Coronary Intervention Versus Coronary Artery Bypass Grafting in Left Main Coronary Artery Disease. Am J Cardiol. 2017;119(12):1949-56.
67. Khan SU, Rahman H, Arshad A, Khan MU, Lekkala M, Yang T, et al. Percutaneous Coronary Intervention Versus Surgery in Left Main Stenosis-A Meta-Analysis and Systematic Review of Randomised Controlled Trials. Heart Lung Circ. 2018;27(2):138-46.
68. Kim JS, Kwon SH, Lee EJ, Yoon YJ. Can Intracapsular Tonsillectomy Be an Alternative to Classical Tonsillectomy? A Meta-analysis. J Int Med Res. 2017;45(3):897-903. doi: 10.1177/0300060517701356. Epub 2017 Apr 18.
69. Kirmani BH, Jones SG, Malaisrie SC, Chung DA, Williams RJ. Limited versus full sternotomy for aortic valve replacement. Cochrane Database Syst Rev. 2017;4(2):CD011793.
70. Klugarova J, Hood V, Bath-Hextall F, Klugar M, Mareckova J, Kelnarova Z. Effectiveness of surgery for adults with hallux valgus deformity: a systematic review. JBI Database System Rev Implement Rep. 2017;15(6):1671-710.
71. Komaei I, Navarra G, Curro G. Three-Dimensional Versus Two-Dimensional Laparoscopic Cholecystectomy: A Systematic Review. J Laparoendosc Adv Surg Tech A. 2017;27(8):790-4.
72. Kong J, Liu P, Fan X, Wen J, Zhang J, Zhen Y, et al. Long-term Outcomes of Paclitaxel-Eluting Versus Sirolimus-Eluting Stent for Percutaneous Coronary Intervention: AMeta-Analysis. J Coll Physicians Surg Pak. 2017;27(7):432-9.
73. Kunath F, Schmidt S, Krabbe LM, Miernik A, Dahm P, Cleves A, et al. Partial nephrectomy versus radical nephrectomy for clinical localised renal masses. Cochrane Database Syst Rev. 2017;5(1):CD012045.
74. Lauridsen SV, Tonnesen H, Jensen BT, Neuner B, Thind P, Thomsen T. Complications and health-related quality of life after robot-assisted versus open radical cystectomy: a systematic review and meta-analysis of four RCTs. Syst Rev. 2017;6(1):150.
75. Li AB, Zhang WJ, Wang J, Guo WJ, Wang XH, Zhao YM. Intramedullary and extramedullary fixations for the treatment of unstable femoral intertrochanteric fractures: a meta-analysis of prospective randomized controlled trials. International Orthopaedics. 2017;41(2):403-13.
76. Li C, Dai Z, Gong Y, Xie B, Wang B. A systematic review and meta-analysis of randomized controlled trials comparing hysteroscopic morcellation with resectoscopy for patients with endometrial lesions. Int J Gynaecol Obstet. 2017;136(1):6-12.
77. Li Y, Yang JJ, Zhu SH, Xu B, Wang L. Long-term efficacy and safety of carotid artery stenting versus endarterectomy: A meta-analysis of randomized controlled trials. PLoS One. 2017;12(7):e0180804.
78. Liu L, Su SW, Sun HY. Safety of Extracranial-Intracranial Arterial Bypass in the Treatment of Moyamoya Disease. J Invest Surg. 2018;31(1):14-23. doi: 10.1080/08941939.2016.1269853. Epub 2017 Jan 6.
79. Locke JA, Noparast M, Afshar K. Treatment of varicocele in children and adolescents: A systematic review and meta-analysis of randomized controlled trials. J Pediatr Urol. 2017;13(5):437-45.
80. Lopez-Cano M, Brandsma HT, Bury K, Hansson B, Kyle-Leinhase I, Alamino JG, et al. Prophylactic mesh to prevent parastomal hernia after end colostomy: a meta-analysis and trial sequential analysis. Hernia. 2017;21(2):177-89.
81. Lu W, Zhu Y, Han Z, Wang X, Wang X, Qiu C. Drug-coated balloon in combination with bare metal stent strategy for de novo coronary artery disease: A PRISMA-compliant meta-analysis of randomized clinical trials. Medicine (Baltimore). 2017;96(12):e6397.
82. Ma T, Zeng C, Pan J, Zhao C, Fang H, Cai D. Remnant preservation in anterior cruciate ligament reconstruction versus standard techniques: a meta-analysis of randomized controlled trials. J Sports Med Phys Fitness. 2017;57(7-8):1014-22.
83. Ma XL, Zhao XW, Ma JX, Li F, Wang Y, Lu B. Effectiveness of surgery versus conservative treatment for lumbar spinal stenosis: A system review and meta-analysis of randomized controlled trials. Int J Surg. 2017;44(7):329-38.
84. Mahmoud AN, Barakat AF, Elgendy AY, Schneibel E, Mentias A, Abuzaid A, et al. Long-Term Efficacy and Safety of Everolimus-Eluting Bioresorbable Vascular Scaffolds Versus Everolimus-Eluting Metallic Stents: A Meta-Analysis of Randomized Trials. Biomed Inform Insights. 2017;9:1178222617697975.(doi):10.1177/1178222617697975. eCollection 2017.
85. Mannu GS, Sudul MK, Bettencourt-Silva JH, Cumber E, Li F, Clark AB, et al. Closure methods of the appendix stump for complications during laparoscopic appendectomy. Cochrane Database Syst Rev. 2017;11(3):CD006437.
86. Marsh N, Webster J, Mihala G, Rickard CM. Devices and dressings to secure peripheral venous catheters: A Cochrane systematic review and meta-analysis. Int J Nurs Stud. 2017;67(doi):12-9.
87. McClure GR, Belley-Cote EP, Jaffer IH, Dvirnik N, An KR, Fortin G, et al. Surgical ablation of atrial fibrillation: a systematic review and meta-analysis of randomized controlled trials. Biomolecules. 2017;7(4).(pii):biom7040079. doi: 10.3390/biom.
88. Menahem B, Vallois A, Alves A, Lubrano J. Prophylactic pelvic drainage after rectal resection with extraperitoneal anastomosis: is it worthwhile? A meta-analysis of randomized controlled trials. Int J Colorectal Dis. 2017;32(11):1531-8.
89. Metcalfe C, Muzaffar J, Daultrey C, Coulson C. Coblation tonsillectomy: a systematic review and descriptive analysis. Eur Arch Otorhinolaryngol. 2017;274(6):2637-47.
90. Mokhles S, Macbeth F, Treasure T, Younes RN, Rintoul RC, Fiorentino F, et al. Systematic lymphadenectomy versus sampling of ipsilateral mediastinal lymph-nodes during lobectomy for non-small-cell lung cancer: a systematic review of randomized trials and a meta-analysis. Eur J Cardiothorac Surg. 2017;51(6):1149-56.
91. Molegraaf M, Kaufmann R, Lange J. Comparison of self-gripping mesh and sutured mesh in open inguinal hernia repair: A meta-analysis of long-term results. Surgery. 2018;163(2):351-60.
92. Montone RA, Niccoli G, De Marco F, Minelli S, D'Ascenzo F, Testa L, et al. Temporal Trends in Adverse Events After Everolimus-Eluting Bioresorbable Vascular Scaffold Versus Everolimus-Eluting Metallic Stent Implantation: A Meta-Analysis of Randomized Controlled Trials. Circulation. 2017;135(22):2145-54.
93. Moore P, Burrage M, Garrahy P, Lim R, McCann A, Camuglia A. Drug-Eluting Stents Versus Coronary Artery Bypass Grafts for Left Main Coronary Disease: A Meta-Analysis and Review of Randomised Controlled Trials. Heart Lung Circ. 2018;27(12):1437-45.
94. Moresoli P, Habib B, Reynier P, Secrest MH, Eisenberg MJ, Filion KB. Carotid Stenting Versus Endarterectomy for Asymptomatic Carotid Artery Stenosis: A Systematic Review and Meta-Analysis. Stroke. 2017;48(8):2150-7.
95. Nairooz R, Saad M, Elgendy IY, Mahmoud AN, Habash F, Sardar P, et al. Long-term outcomes of provisional stenting compared with a two-stent strategy for bifurcation lesions: a meta-analysis of randomised trials. Heart. 2017;103(18):1427-34.
96. Osland E, Yunus RM, Khan S, Memon B, Memon MA. Weight Loss Outcomes in Laparoscopic Vertical Sleeve Gastrectomy (LVSG) Versus Laparoscopic Roux-en-Y Gastric Bypass (LRYGB) Procedures: A Meta-Analysis and Systematic Review of Randomized Controlled Trials. Surg Laparosc Endosc Percutan Tech. 2017;27(1):8-18.
97. Osland E, Yunus RM, Khan S, Memon B, Memon MA. Changes in Non-Diabetic Comorbid Disease Status Following Laparoscopic Vertical Sleeve Gastrectomy (LVSG) Versus Laparoscopic Roux-En-Y Gastric Bypass (LRYGB) Procedures: a Systematic Review of Randomized Controlled Trials. Obes Surg. 2017;27(5):1208-21.
98. Osland E, Yunus RM, Khan S, Memon B, Memon MA. Diabetes improvement and resolution following laparoscopic vertical sleeve gastrectomy (LVSG) versus laparoscopic Roux-en-Y gastric bypass (LRYGB) procedures: a systematic review of randomized controlled trials. Surg Endosc. 2017;31(4):1952-63.
99. Palmerini T, Serruys P, Kappetein AP, Genereux P, Riva DD, Reggiani LB, et al. Clinical outcomes with percutaneous coronary revascularization vs coronary artery bypass grafting surgery in patients with unprotected left main coronary artery disease: A meta-analysis of 6 randomized trials and 4,686 patients. Am Heart J. 2017;190(doi):54-63.
100. Patel SV, Zhang L, Chadi SA, Wexner SD. Prophylactic mesh to prevent parastomal hernia: a meta-analysis of randomized controlled studies. Tech Coloproctol. 2017;21(1):5-13.
101. Patterson T, Currie P, Patterson S, Patterson P, Meek C, McMaster R. A systematic review and meta-analysis of the post-operative adverse effects associated with mosquito net mesh in comparison to commercial hernia mesh for inguinal hernia repair in low income countries. Hernia. 2017;21(3):397-405.
102. Pedziwiatr M, Malczak P, Mizera M, Witowski J, Torbicz G, Major P, et al. There is no difference in outcome between laparoscopic and open surgery for rectal cancer: a systematic review and meta-analysis on short- and long-term oncologic outcomes. Techniques in Coloproctology. 2017;21(8):595-604.
103. Pundir J, Omanwa K, Kovoor E, Pundir V, Lancaster G, Barton-Smith P. Laparoscopic Excision Versus Ablation for Endometriosis-associated Pain: An Updated Systematic Review and Meta-analysis. J Minim Invasive Gynecol. 2017;24(5):747-56.
104. Putzu A, Gallo M, Martino EA, Ferrari E, Pedrazzini G, Moccetti T, et al. Coronary artery bypass graft surgery versus percutaneous coronary intervention with drug-eluting stents for left main coronary artery disease: A meta-analysis of randomized trials. Int J Cardiol. 2017;241(1):142-8.
105. Pynnonen M, Brinkmeier JV, Thorne MC, Chong LY, Burton MJ. Coblation versus other surgical techniques for tonsillectomy. Cochrane Database Syst Rev. 2017;8:CD004619.
106. Qian C, Feng H, Cao J, Wei B, Wang Y. Meta-Analysis of Randomized Control Trials Comparing Drug-Eluting Stents Versus Coronary Artery Bypass Grafting for Significant Left Main Coronary Narrowing. Am J Cardiol. 2017;119(9):1338-43.
107. Redden MD, Chin TY, van Driel ML. Surgical versus non-surgical management for pleural empyema. Cochrane Database Syst Rev. 2017;3(6):CD010651.
108. Rezende FC, Moraes VY, Franciozi CE, Debieux P, Luzo MV, Belloti JC. One-incision versus two-incision techniques for arthroscopically assisted anterior cruciate ligament reconstruction in adults. Cochrane Database Syst Rev. 2017;12(10):CD010875.
109. Ricci C, Casadei R, Taffurelli G, Pacilio CA, Beltrami D, Minni F. Is pancreaticogastrostomy safer than pancreaticojejunostomy after pancreaticoduodenectomy? A meta-regression analysis of randomized clinical trials. Pancreatology. 2017;17(5):805-13.
110. Ryosa A, Laimi K, Aarimaa V, Lehtimaki K, Kukkonen J, Saltychev M. Surgery or conservative treatment for rotator cuff tear: a meta-analysis. Disabil Rehabil. 2017;39(14):1357-63.
111. Saber AA, Shoar S, Almadani MW, Zundel N, Alkuwari MJ, Bashah MM, et al. Efficacy of First-Time Intragastric Balloon in Weight Loss: a Systematic Review and Meta-analysis of Randomized Controlled Trials. Obes Surg. 2017;27(2):277-87.
112. Sajid MS, Rathore MA, Sains P, Singh KK. A systematic review of clinical effectiveness of wound edge protector devices in reducing surgical site infections in patients undergoing abdominal surgery. Updates Surg. 2017;69(1):21-8.
113. Sakran JV, Mylonas KS, Gryparis A, Stawicki SP, Burns CJ, Matar MM, et al. Operation versus antibiotics-The "appendicitis conundrum" continues: A meta-analysis. Journal of Trauma and Acute Care Surgery. 2017;82(6):1129-37.
114. Sardar P, Chatterjee S, Aronow HD, Kundu A, Ramchand P, Mukherjee D, et al. Carotid Artery Stenting Versus Endarterectomy for Stroke Prevention: A Meta-Analysis of Clinical Trials. J Am Coll Cardiol. 2017;69(18):2266-75.
115. Sardar P, Giri J, Elmariah S, Chatterjee S, Kolte D, Kundu A, et al. Meta-Analysis of Drug-Eluting Stents Versus Coronary Artery Bypass Grafting in Unprotected Left Main Coronary Narrowing. Am J Cardiol. 2017;119(11):1746-52.
116. Sarode D, Bari DA, Cain AC, Syed MI, Williams AT. The benefit of silicone stents in primary endonasal dacryocystorhinostomy: a systematic review and meta-analysis. Clin Otolaryngol. 2017;42(2):307-14.
117. Scheuermann U, Niebisch S, Lyros O, Jansen-Winkeln B, Gockel I. Transabdominal Preperitoneal (TAPP) versus Lichtenstein operation for primary inguinal hernia repair - A systematic review and meta-analysis of randomized controlled trials. BMC Surg. 2017;17(1):55.
118. Schuurmans J, Goslings JC, Schepers T. Operative management versus non-operative management of rib fractures in flail chest injuries: a systematic review. Eur J Trauma Emerg Surg. 2017;43(2):163-8.
119. Shaikh FM, Stewart PM, Walsh SR, Davies RJ. Laparoscopic peritoneal lavage or surgical resection for acute perforated sigmoid diverticulitis: A systematic review and meta-analysis. Int J Surg. 2017;38(2):130-7.
120. Shangguan L, Ning GZ, Tang Y, Wang Z, Luo ZJ, Zhou Y. Discover cervical disc arthroplasty versus anterior cervical discectomy and fusion in symptomatic cervical disc diseases: A meta-analysis. PLoS One. 2017;12(3):e0174822.
121. Sharma SP, Dahal K, Khatra J, Rosenfeld A, Lee J. Percutaneous coronary intervention vs coronary artery bypass grafting for left main coronary artery disease? A systematic review and meta-analysis of randomized controlled trials. Cardiovasc Ther. 2017;35(3):820-7. doi: 10.1002/dc.23716. Epub 2017 Mar 31.
122. Sharma SP, Sangha RS, Dahal K, Krishnamoorthy P. The role of empiric superior vena cava isolation in atrial fibrillation: a systematic review and meta-analysis of randomized controlled trials. J Interv Card Electrophysiol. 2017;48(1):61-7.
123. Sun P, Cheng X, Deng S, Hu Q, Sun Y, Zheng Q. Mesh fixation with glue versus suture for chronic pain and recurrence in Lichtenstein inguinal hernioplasty. Cochrane Database Syst Rev. 2017;2(3):CD010814.
124. Tan C, Ocampo O, Ong R, Tan KS. Comparison of one stage laparoscopic cholecystectomy combined with intra-operative endoscopic sphincterotomy versus two-stage pre-operative endoscopic sphincterotomy followed by laparoscopic cholecystectomy for the management of pre-operatively diagnosed patients with common bile duct stones: a meta-analysis. Surg Endosc. 2018;32(2):770-8.
125. Tan CC, Wang HF, Ji JL, Tan MS, Tan L, Yu JT. Endovascular Treatment Versus Intravenous Thrombolysis for Acute Ischemic Stroke: a Quantitative Review and Meta-Analysis of 21 Randomized Trials. Mol Neurobiol. 2017;54(2):1369-78.
126. Tang Q, Shang P, Zheng G, Xu HZ, Liu HX. Extramedullary versus intramedullary femoral alignment technique in total knee arthroplasty: a meta-analysis of randomized controlled trials. J Orthop Surg Res. 2017;12(1):82.
127. Tong MJ, Tang Q, Wang CG, Xiang GH, Chen Q, Xu HZ, et al. Efficacy of Using Intermediate Screws in Short-Segment Fixation for Thoracolumbar Fractures: A Meta-Analysis of Randomized Controlled Trials. Transl Lung Cancer Res. 2017;6(5):588-99. doi: 10.21037/tlcr.2017.08.06.
128. Tse F, Yuan Y, Moayyedi P, Leontiadis GI, Barkun AN. Double-guidewire technique in difficult biliary cannulation for the prevention of post-ERCP pancreatitis: a systematic review and meta-analysis. Endoscopy. 2017;49(1):15-26.
129. Upadhaya S, Baniya R, Madala S, Subedi SK, Khan J, Velagapudi RK, et al. Drug-eluting stent placement versus coronary artery bypass surgery for unprotected left main coronary artery disease: A meta-analysis of randomized controlled trials. J Card Surg. 2017;32(2):70-9.
130. van Agteren JE, Hnin K, Grosser D, Carson KV, Smith BJ. Bronchoscopic lung volume reduction procedures for chronic obstructive pulmonary disease. Cochrane Database Syst Rev. 2017;2(doi):CD012158.
131. van der Ploeg JM, van der Steen A, Zwolsman S, van der Vaart CH, Roovers J. Prolapse surgery with or without incontinence procedure: a systematic review and meta-analysis. BJOG. 2018.
132. Veldman HD, Heyligers IC, Grimm B, Boymans TA. Cemented versus cementless hemiarthroplasty for a displaced fracture of the femoral neck: a systematic review and meta-analysis of current generation hip stems. Bone Joint J. 2017;99-B(4):421-31.
133. Vellayappan BA, Soon YY, Ku GY, Leong CN, Lu JJ, Tey JC. Chemoradiotherapy versus chemoradiotherapy plus surgery for esophageal cancer. Cochrane Database Syst Rev. 2017;8(doi):CD010511.
134. Vidale S, Agostoni E. Endovascular Treatment of Ischemic Stroke: An Updated Meta-Analysis of Efficacy and Safety. Vasc Endovascular Surg. 2017;51(4):215-9.
135. Wang CH, Zhang SY, Jin XF. Complete revascularization versus culprit-only revascularization in ST-segment elevation myocardial infarction and multivessel disease patients undergoing primary percutaneous coronary intervention: A meta-analysis and trial sequential analysis. International Journal of Cardiology. 2017;228(7):844-52.
136. Wang H, Man L, Li G, Huang G, Liu N, Wang J. Meta-Analysis of Stenting versus Non-Stenting for the Treatment of Ureteral Stones. PLoS One. 2017;12(1):e0167670.
137. Wang WW, Dong BC. Comparison on effectiveness of trans-septal suturing versus nasal packing after septoplasty: a systematic review and meta-analysis. Eur Arch Otorhinolaryngol. 2017;274(11):3915-25.
138. Wang X, He JJ, Chen X, Yang QQ. Stenting as a bridge to resection versus emergency surgery for left-sided colorectal cancer with malignant obstruction: A systematic review and meta-analysis. International Journal of Surgery. 2017;48(suppl_3):64-8.
139. Wang XC, Zhang D, Yang ZX, Gan JX, Yin LN. Mesh reinforcement for the prevention of incisional hernia formation: a systematic review and meta-analysis of randomized controlled trials. J Surg Res. 2017;209(doi):17-29.
140. Wang Y, Wen M, Zhou J, Chen Y, Zhang Q. Coronary artery bypass grafting versus percutaneous coronary intervention in patients with noninsulin treated type 2 diabetes mellitus: A meta-analysis of randomized controlled trials. Diabetes Metab Res Rev. 2018;34(1):327-44. doi: 10.1111/bju.14018. Epub 2017 Nov 24.
141. Woltz S, Krijnen P, Schipper IB. Plate Fixation Versus Nonoperative Treatment for Displaced Midshaft Clavicular Fractures: A Meta-Analysis of Randomized Controlled Trials. J Bone Joint Surg Am. 2017;99(12):1051-7.
142. Wu X, Liu Q, Zhang R, Wang W, Gao Y. Therapeutic efficacy and safety of laparoscopic surgery versus microsurgery for varicocele of adult males: A meta-analysis. Medicine (Baltimore). 2017;96(34):e7818.
143. Xie L, Zhao ZG, Zhang SJ, Hu YB. Percutaneous vertebroplasty versus conservative treatment for osteoporotic vertebral compression fractures: An updated meta-analysis of prospective randomized controlled trials. Int J Surg. 2017;47(3):25-32.
144. Xu XL, Liu XD, Liang M, Luo BM. Radiofrequency Ablation versus Hepatic Resection for Small Hepatocellular Carcinoma: Systematic Review of Randomized Controlled Trials with Meta-Analysis and Trial Sequential Analysis. Radiology. 2018;287(2):461-72.
145. Yang L, Zhang B, Xing G, Du J, Yang B, Yuan Q, et al. Neoadjuvant chemotherapy versus primary debulking surgery in advanced epithelial ovarian cancer: A meta-analysis of peri-operative outcome. PLoS One. 2017;12(10):e0186725.
146. Yang S, Chen C, Wang H, Wu Z, Liu L. A systematic review of unilateral versus bilateral percutaneous vertebroplasty/percutaneous kyphoplasty for osteoporotic vertebral compression fractures. Acta Orthop Traumatol Turc. 2017;51(4):290-7.
147. Zhang LL, Zhang Y, Ma X, Liu Y. Multiple cannulated screws vs. dynamic hip screws for femoral neck fractures : A meta-analysis. Orthopade. 2017;46(11):954-62.
148. Zhang S, Lan Z, Zhang J, Chen Y, Xu Q, Jiang Q, et al. Duct-to-mucosa versus invagination pancreaticojejunostomy after pancreaticoduodenectomy: a meta-analysis. Oncotarget. 2017;8(28):46449-60.
149. Zhang W, Li G, Chen YL. Should T-Tube Drainage be Performed for Choledocholithiasis after Laparoscopic Common Bile Duct Exploration? A Systematic Review and Meta-Analysis of Randomized Controlled Trials. Surg Laparosc Endosc Percutan Tech. 2017:415–23.
150. Zhao JG, Wang J, Meng XH, Zeng XT, Kan SL. Surgical interventions to treat humerus shaft fractures: A network meta-analysis of randomized controlled trials. PLoS One. 2017;12(3):e0173634.
151. Zhao S, Xu CY, Zhu AR, Ye L, Lv LL, Chen L, et al. Comparison of the efficacy and safety of 3 treatments for patients with osteoporotic vertebral compression fractures: A network meta-analysis. Medicine (Baltimore). 2017;96(26):e7328.
152. Zhao X, Cui N, Wang X, Cui Y. Surgical strategies in the treatment of chronic pancreatitis: An updated systematic review and meta-analysis of randomized controlled trials. Medicine (Baltimore). 2017;96(9):e6220.
153. Zhao XW, Ma JX, Ma XL, Li F, He WW, Jiang X, et al. Interspinous process devices(IPD) alone versus decompression surgery for lumbar spinal stenosis(LSS): A systematic review and meta-analysis of randomized controlled trials. Int J Surg. 2017;39(doi):57-64.
154. Zhu Q, Xu X, Yang X, Chen X, Wang L, Liu C, et al. Intramedullary nails versus sliding hip screws for AO/OTA 31-A2 trochanteric fractures in adults: A meta-analysis. Int J Surg. 2017;43(8):67-74.
155. Maher C, Baessler K, Glazener CM, Adams EJ, Hagen S. Surgical management of pelvic organ prolapse in women. Cochrane Database Syst Rev. 2017(3):CD004014.
